# High quality nursing based on childlike interest in children with cleft lip and palate: application assessment after operation

**DOI:** 10.1186/s12903-021-01893-6

**Published:** 2021-11-23

**Authors:** Yi Peng, Xiaoyan Hao, Yuan Guo, Xueqin Zhang, Yang Li, Yanmei Ma, Juan Wang

**Affiliations:** Department of Plastic and Cosmetic Maxillofacial Surgery, First Affiliated Hospital of Xi’an Jiaotong University, No. 277 Yanta West Road, Xi’an, 710061 Shaanxi China

**Keywords:** High quality nursing based childlike interest, Children with cleft lip and palate after operation, Application value

## Abstract

**Purpose:**

The aim of this study was to assess the effect of high-quality nursing based on the concept of childlike interest in children with cleft lip and palate following operation on healing time, degree of pain, psychological state, quality of life, and the occurrence of complications.

**Methods:**

A series of 62 children with cleft lip and palate was treated in our hospital from January 2019 to March 2021. The patients were randomly divided into observation group (31 cases, given high-quality nursing based on childlike interest) and control group (31 cases, given routine nursing intervention). The healing time and hospital stay of the two groups were recorded. The degree of pain, psychological state and quality of life of the two groups before and after intervention were compared, and the occurrence of complications was closely monitored.

**Results:**

Compared with the control group, the healing time and hospital stay of the study group were significantly shorter after the intervention (P < 0.05). Before the intervention, no significant difference was identified in pain score between the two groups (P < 0.05), after the intervention, however, the pain score of the study group was significantly lower compared with the control group (P < 0.05). Self-Rating Depression Scale (SDS) and Self-Rating Anxiety Scale (SAS) scores of the two groups were comparable before intervention (P > 0.05), while after intervention the SDS and SAS scores of the two groups were lower than those before treatment. Compared with the control group, the SDS and SAS scores of the study group were remarkably lower (P < 0.05). Before the intervention, the quality of life scores of the two groups were comparable (P > 0.05), while after the intervention, the scores of quality of life in the two groups were associated with lower outcomes. Compared with the control group, the scores of quality of life in the study group were significant lower (P < 0.05). After the intervention, there were evident fewer incidence of complications in the study group compared to the control group (P < 0.05).

**Conclusions:**

High quality nursing based on childlike interest exerted beneficial outcomes in terms of shortening the healing time and hospital stay, reducing the degree of pain and complications, as well as improving the psychological state and quality of life of children harboring cleft lip and palate. Additionally, its high safety feature contributes to the wide application for clinical practice.

## Introduction

Cleft lip and palate, a common congenital malformation disease in clinical pediatrics, affects the facial morphology, structure and function of children. It also brings negative impact on the physical and mental health of children, and causes both financial and psychological burden to children’s families [1, 2]. Children with cleft lip and palate are usually accompanied by different degrees of bone malformation or even defect, which lead to occlusal disorder and language dysfunction. In addition, children prone to anxiety, inferiority and other adverse psychological [3, 4]. Currently, children harboring cleft lip and palate should seek surgical intervention. And the main purpose is to have plastic surgery. Good treatment effect could generally be obtained by plastic surgery among children aged 1–3 years old. However, considering children in remote areas fail to seek early intervention, there is no unified standard for the operation time [5]. Most children may experience different degrees of pain after treatment due to the traumatic operation of plastic surgery. Children are too young for strong cognitive function easy, hence anxiety, fear and other negative emotions may occur. It is not conducive to the postoperative treatment and nursing, and has a negative impact on the prognosis of children.

High quality nursing based on the concept of childlike interest is performed to integrate various nursing measures with childlike interest, improve the quality of nursing and the design in line with children’s cognitive interest, with an attempt to improve the compliance of children and improve the nursing effect[6]. Currently, the concept of childlike interest has been applied to respiratory care in clinic [6], while it is rarely applied in cleft lip and palate.

Herein, the present study was carried out to explore the application value of the concept of childlike interest among children after cleft lip and palate surgery by evaluating the healing time, degree of pain, psychological state, quality of life, occurrence of complications.

## Materials and methods

### Participants

A series of 62 cases of children with cleft lip and palate after operation was selected in our hospital from January 2019 to March 2021. All participants were divided into study group and control group based on the random number table method, 31 cases in each group. In study group, there involved 18 males and 13 females, aged 3–5 years old, average (4.12 ± 1.87) years old of age; location: 18 cases with left cleft lip and palate, 13 cases with right cleft lip and palate; residence: 14 in rural areas and 17 in urban areas; educational background of parents: 12 cases of junior high school and below, 19 cases of senior high school and above. In control group, there were 17 males and 14 females, aged 3–5 years old, average (4.09 ± 1.82) years old of age; location: 19 cases with left cleft lip and palate, 12 cases with right cleft lip and palate; residence: 13 cases in rural areas and 18 in urban areas; educational background of parents: 11 cases of junior high school and below, 20 cases of senior high school and above. The general data of the two groups were comparable (P > 0.05). The study was approved by the Ethics Committee of First Affiliated Hospital of Xi’an Jiaotong University. The current research program meets the requirements of the declaration of Helsinki of the World Medical Association.

### Sample size calculation

The sample size was calculated with this method [7]: n_c_ = (*µ*_1−α/2_*µ*_1−β_)^2^s^2^(1 + 1/k)/(*µ*_t_ − µ_c_)^2^.

n_c_ was the number of the control group. *µ*_t_ was the assumed mean value of the study group, *µ*_c_ was the assumed mean value of the control group, while K was the ratio of the two groups. Take α = 0.05, β = 0.01, assumed a 15% loss of follow-up rate for the study subjects, the sample size of each group in this study was determined to be [n = 36 × 1/(1 − 0.15) = 30.59 ≈ 31].

### Selection criteria

In order to be included in the current study, the following inclusion criteria should be met: all children met the diagnostic criteria of cleft lip and palate in oral and maxillofacial surgery (6th Edition), who were congenital cleft lip and palate; patients with the normal intelligence to communicate normally; patients agreed to receive cleft lip and palate plastic surgery in our hospital and signed the informed consent; good compliance.

Exclusion criteria were parents unwilling to participate in the study; age < 3 years-old, or > 5 years-old; combined with congenital substantive organ dysfunction; congenital mental retardation; children without immediate family care; complicated with serious diseases; children who could not express their pain level.

## Methods

The control group was given routine nursing methods, including drug knowledge publicity, individualized treatment of physical symptoms, basic life nursing, health education of family members, diet guidance, basic nursing and morning and evening nursing.

The observation group was given the concept of childlike interest on the basis of the above nursing. A high-quality nursing group with the concept of childlike interest was established, which was composed of one head nurse in pediatrics, one national second-class psychologist, two supervisor nurses, two senior nurses and four nurses. All of them participated in the assessment of knowledge about cleft lip and palate, and they could be enrolled after the assessment. The head nurse was the group leader, responsible for quality supervision during the whole research process. A supervisor nurse served as the deputy team leader, responsible for coordinating the work of all posts, allocating resources and distributing the adjustment plan of the team leader. The other supervisor nurse was responsible for training all nursing stuff. The psychologist provided guidance on the psychology-related issues involved throughout the study. The other nurses performed pre-intervention propaganda, behavior guidance, situational drills and data collection. In addition, the chief physician of the department was invited as a consultant, and the team members were coordinated to design the intervention process, which was submitted to the nursing department and medical Department of our hospital for review, to determine the final responsibility of each post, intervention content and intervention process.

The specific contents were as follows: (1) education of childlike interest. The team members mastered and understood the emotional needs and emotional state of the children through induction interview based on childlike interest, and communicated with the family members of the children. According to the interview results and the information provided by the family members, the personality characteristics of the children were evaluated. If the children are introverted, the basic precautions after cleft lip and palate repair will be shown to the children through the hand-painted cartoon animation for attention. The method can have childlike interest and favor to establish a good relationship with patients, with an attempt to promote cooperation with the medical treatment measures. The team members also popularized the knowledge of cleft lip and palate to the children and their families through animation short films and cartoons, and integrate the basic situation and self-care skills after repair into the animation full of childlike interest, to promote their understanding of situation after repair. The procedure was believed benefit for psychological construction, to overcome passive emotions and establish confidence in treatment. In addition, the group members should guide the children and their families, emphasize and explain the cautions after the repair to the children in the way of childlike interest. Family members should be advised to relax and return to the state of innocence in the process of explanation to produce resonance. Meanwhile, the group members should guide the children to put forward the corresponding questions, and the family members should answer them in time. Through guiding family members to participate in nursing intervention and children’s education, we can promote them to fully understand the disease and cooperate with the implementation of the nursing program [8]. Childlike scene drill. According to the children’s knowledge of cleft lip and palate, combined with their own experience, the team members designed the scene of childlike interest for on-site drill. In the process of drill, the patients were focused and introduced the specific situation and relevant precautions after repair, with attempts to encourage them build confidence in rehabilitation. The team members should patiently ask the children about the strategies to reduce pain in the process of rehabilitation, and give affirmation, encouragement and praise to the correct measures, and correct the wrong measures. The family members should use appropriate language to encourage the children to adopt the correct analgesia strategy, and at the same time use the way of physical comfort to help the children put forward relevant measures confidently [6]. In the whole process of postoperative rehabilitation, the team members should ventilate and disinfect the ward regularly every day, and take insulation measures, and pay attention to adjust the appropriate temperature and humidity.

### Observation indexes

The healing time and hospital stay of the two groups were recorded [9]. The pain degree of the children before and after the intervention was compared and evaluated by the Children’s Hospital of Eastern Ontario Pain Scale (CHEOPS)[10]. The scale recorded pain based on crying, language, facial expression, posture, touching wound and leg movement. The total score was the sum of each item, with a score of 0–13. Higher score represents high level of pain [9]. Psychological status: the psychological status of the two groups were compared before and after the intervention, and evaluated by Self-Rating Depression Scale (SDS) [8] and Self-Rating Anxiety Scale (SAS) [11, 12]. There were 20 items in SDS and SAS, and 4-level scoring method was used. The integral part of each item multiplied by 1.25 was the final total score, in which mild depression or anxiety was 50–59 points, moderate depression or anxiety was 60–69 points, and severe depression or anxiety was more than 70 points. (4) Quality of life: the children’s quality of life scale (PedsQLTM 4.0) [13] was used to evaluate quality of life before and after treatment. This study mainly involved the 2–4 years old and 5–7 years old parts of the scale. The scale included four dimensions, including physiological function, role function, social function and emotional function, with a total of 23 questions, with a score of 0–4 for each question and a total score of 0-100. The higher the score, the higher the quality of life. (5) Complications: detailed records in 3 months in children with infection, laryngeal edema, cleft relapse, aspiration, fever and other complications.

#### Statistical analysis

Statistical analysis was carried out using SPSS 21.0 software to analyze the data. The healing time and hospital stay was measured by using $\bar{x}$ ± s and t test. The counting data were expressed as percentage (%) and chi square was used through χ^2^ inspection. A P<0.05 represented statistical significance.

## Results

### Comparison of healing time and hospital stay between the two groups

After the intervention, the healing time and hospital stay of the study group were significantly shorter compared with the control group (P < 0.001, respectively), as laid out in Table 1.

**Table 1:** Comparison of healing time and hospital stay between the two groups ($\bar{x}$  ± s, d)

Group (n)	Healing time	Hospital stay
Study group (31)	25.02 ± 2.93^*#*^	9.68 ± 1.02^*#*^
Control group (31)	33.12 ± 2.76	14.67 ± 1.23
t value	− 11.204	− 17.387
P value	< 0.001	< 0.001

^#^P < 0.05: the comparison of the control group after the intervention

## Comparison of pain degree between the two groups

Before the intervention, there was no significant difference in pain score between the two groups (P = 0.375), while after the intervention, the pain degree score of the study group was significantly lower compared with the control group (P < 0.001), as shown in Table 2.

**Table 2:** Comparison of pain degree between the two groups ($\bar{x}$ ± s, points)

Group (n)	Before intervention	After intervention
Study group (31)	7.67 ± 0.23	2.07 ± 0.23*^#^
Control group (31)	7.62 ± 0.21	5.32 ± 0.27*
t value	0.894	− 50.018
P value	0.375	< 0.001

*P < 0.05: the comparison of the same group before treatment; ^#^P < 0.05: the comparison of the control group after treatment

### Comparison of psychological state between the two groups

Before intervention, SDS and SAS scores of the two groups were comparable (P = 0.755, 0.317, respectively). After the intervention, the SDS and SAS scores of the two groups were lower than those before treatment. Compared with the control group, the SDS and SAS scores of the study group were significantly lower (P < 0.001, respectively), as laid out in Table 3.

**Table 3:** Comparison of psychological state between the two groups ($\bar{x}$ ± s, points)

Items	Study group (n = 31)	Control group (n = 31)	t value	P value
*SDS*				
Before intervention	56.11 ± 3.49	55.83 ± 3.53	0.314	0.755
After intervention	31.82 ± 3.08*^#^	45.82 ± 3.83*	− 15.860	< 0.001
*SAS*				
Before intervention	58.76 ± 3.23	57.76 ± 4.48	1.008	0.317
After intervention	31.56 ± 3.02*^#^	43.29 ± 3.07*	− 15.166	< 0.001

*P < 0.05: the comparison of the same group before treatment; ^#^P < 0.05: the comparison of the control group after treatment

### Comparison of quality of life between the two groups

Before the intervention, the scores of quality of life of the two groups were comparable (P > 0.05, respectively). After the intervention, the scores of quality of life in the two groups were lower than those before treatment. The scores of quality of life in the study group were significantly lower compared to the control group (P < 0.001, respectively), as shown in Table 4.

**Table 4:** Comparison of quality of life between the two groups ($\bar{x}$  ± s, points)

Items	Study group (n = 31)	Control group (n = 31)	t value	P value
*Physiological function*				
Before intervention	16.87 ± 1.23	16.86 ± 1.25	0.032	0.975
After intervention	26.98 ± 1.32*^#^	18.76 ± 1.26*	25.080	< 0.001
*Role function*				
Before intervention	10.92 ± 1.26	10.87 ± 1.23	0.158	0.875
After intervention	17.02 ± 1.27*^#^	12.14 ± 1.07*	16.361	< 0.001
*Social function*				
Before intervention	8.87 ± 1.02	8.83 ± 1.05	0.152	0.880
After intervention	16.82 ± 1.09*^#^	11.98 ± 1.01*	18.135	< 0.001
*Emotional function*				
Before intervention	8.76 ± 1.01	8.71 ± 0.98	0.198	0.844
After intervention	16.91 ± 1.13*^#^	12.34 ± 1.06*	16.423	< 0.001

*P < 0.05: the comparison of the same group before treatment; ^#^P < 0.05: the comparison of the control group after treatment

### Comparison of complications between the two groups

After the intervention, there were significantly fewer incidence of complications in the study group compared to the control group (P = 0.023), as shown in Table 5.

**Table 5:** Comparison of complications between the two groups

Group (n)	Infection	Laryngeal edema	Cleft relapse	Aspiration	Fever	Total incidence
Study group (31)	0	1	0	0	0	1 (3.23)^*#*^
Control group (31)	1	3	0	2	1	7 (22.58)
t value						5.167
P value						0.023

^#^P < 0.05: the comparison of the control group after the intervention

## Discussion

The results of this study showed that compared with conventional nursing, high quality nursing based on the concept of childlike interest could reduce the healing time and hospital stay time, relieve the post-operative pain to some extent. Also, the SDS and SAS scores in the study group were significantly lower than the control group. Furthermore, the incidence of complications in the study group was significantly lower.

Cleft lip and palate is acknowledged as one of the common birth defects in clinic. The birth defect rate worldwide is 1.5/10,000, with cleft lip and palate varying greatly with different races, regions and economic conditions [14]. The incidence rate of cleft lip and palate in Asian population accounts for 17.6–18.1 per 10,000, which is higher than the average level in the world (9.9 per million) [15, 16]. The maxillary arch of children with cleft lip and palate is lower than that of normal people because of crowded maxillary dentition and 3d growth and development disorder of maxilla [3]. Dysplasia of face in children with cleft lip and palate will cause adverse psychology and affect their normal social interaction. Early intervention should be sought in order to recover the facial morphology and physiological function of children harboring cleft lip and palate, and to reduce the adverse psychological impact on children and their families. The most important treatment strategy for cleft lip and palate is to carry out surgical repair and orthodontic treatment as soon as possible [3, 17]. Cannavale et al. [17] pointed out that orthodontic treatment can correct maxillary premolar-molar displacement in permanent dentition, and early treatment can prevent molar-premolar displacement. Isola et al. [4] found that the use of an orthodontic functional appliance determined a significant improvement, at 24 months, in many TMJ signs and symptoms in patients affected by JIA and with TMJ disorders. This study suggests that children and adolescents with JIA which presented either unilateral or bilateral moderate to severe TMJ involvement, if not treated, should be develop severe disturbances during growing. Liu et al. [18] suggested that oral intervention is a nice choice to maintain periodontal mucosa health in orthodontic patients, and the effect of this measure increases with the time goes on. Fu et al. [19] believed that effective nursing intervention in orthodontic patients can reduce anxiety, depression and other negative psychological generation, reduce the degree of pain. However, due to the young age of the children and the lack of knowledge about cleft lip and palate repair of their families, most of the children could suffer from serious pain after the surgery, which seriously affects the prognosis. In addition, considering their immature psychological development and poor understanding and cognitive ability of knowledge, routine nursing based on oral education is insufficient for children’s physical and mental health. It is therefore difficult to achieve the ideal intervention effect, and even lead to resistance of children and their families, affecting the relationship between nurses and patients. Previous studies revealed that Early orthodontic treatment can effectively improve the dental arch relationship, optimize the health status of alveolar bone and improve chewing efficiency in children with cleft lip and palate, with high safety [3, 4]. Herein, the high-quality nursing methods are needed for children with cleft lip and palate. Focus has been laid in terms of the methods for guiding children to standardize postoperative behavior, effectively avoiding complications, and reducing the psychological stress reaction caused by surgical trauma.

As the implementation of humanized medical treatment, the concept of high quality nursing strategies has been carried out in depth and widely [20]. According to the cognitive level and psychological characteristics of children, this study introduced the idea of childlike interest in high-quality nursing approaches, established psychological communication channels between nurses and patients through childlike communication. It contributed to the fear elimination of white coat, and the psychological distance between nurses and patients was closer. The strategy also promoted the empathy and collision of understanding in nurse-patient communication. It was beneficial for children’s cognition and attention to the operation and the post operation behavior standard and cooperation were promoted. Meanwhile, the nursing staff and their families should stand in the position of the children, help them to understand the disease, and help the children master the skills of coping with the disease to the greatest extent. According to Augsornman et al. [21], active and effective nursing strategy in the lip and palate cleft surgery can effectively improve the patients’ satisfaction and reduce the occurrence of adverse nursing events. Similarly, Gong et al. [22] confirmed that effective nursing can improve the level of nursing care in children with cleft lip and palate, thus improving the therapeutic effect. Based on our results, the healing time and hospital stay of the study group were shorter than that of the control group, suggesting that the high-quality nursing guided by childish interest idea could shorten the wound healing time and the hospital stay in the children after lip and palate cleft operation. This may attribute to the fact that the high-quality nursing based on childlike interest idea showed the children and their families the relevant knowledge of cleft lip and palate through cartoon short films and comics, which may help them understand the situation after the repair and cooperate with the nursing work. Thus, the prognosis time and hospital stay of the children were shortened.

Pain has become the fifth vital sign apart from body temperature, pulse, breath and blood pressure, and has been paid more attention recently. Postoperative pain is a complex physiological and psychological response to tissue injury and repair process, and it is a pivotal issue for patients after surgery [23]. Therefore, our study monitored the pain degree of children after cleft lip and palate operation. It was showed that the pain degree score of the study group was lower than that of the control group, suggesting that the high-quality nursing guided by childish interest idea could reduce the pain degree in the children after cleft lip and palate operation. This is mainly because the members of the high-quality nursing groups with childlike interest idea patiently and carefully asked the children about the measures to alleviate pain during the recovery process, affirmed and praised the correct measures, and guided the wrong analgesic measures to be converted into the correct ones. Meanwhile, the patients were pacified with body language to put forward or implement the correct analgesic measures.

Previous study [24] documented that the psychological stress reaction can aggravate the discomfort of the body, and the increased pain can also affect the psychology and then aggravate the adverse psychological stress response. Depression, fear, anxiety and other adverse psychological reactions are not conducive to incision healing and recovery time, and even lead to hormone secretion disorders, which has negative impact on the stability of postoperative vital signs, and increases postoperative complications. Based on our results, SDS and SAS scores of the study group were lower than those in the control group, indicating that the high-quality nursing guided by the concept of childish interest could improve the psychological state of the children after cleft lip and palate. In this study, the nursing staff judged and recorded the children’s personality characteristics before surgery, and sought corresponding interventions according to the children’s personality characteristics. On the other hand, the nursing staff guided the family members to learn the childlike language so that they can display the related knowledge of the disease with the childlike language expression, which was child-oriented. The above measures promoted the understanding of the disease and the coordination between the nurses, parents and patients. Most importantly, it reduced the children’s anxiety, depression and other negative psychology, which could reduce the feeling of pain and was conducive to the recovery.

According to Li et al. [25], routine nursing and effective targeted nursing were performed in 52 children with cleft lip and palate using random digital table. The results showed that effective targeted nursing improved the psychological state, the quality of life and the satisfaction of children. Our results demonstrated that the quality of life score of the study group was lower than that of the control group, suggesting that the quality of life of the children after cleft lip and palate surgery could be improved by using the high-quality nursing guided by childlike interest idea. Because through the scene drill of childlike interest in the high-quality nursing, the children’s self-protection awareness was improved, the awareness of behavior standard and risk avoidance measures was deepened. Moreover, during the practice, nurses were able to closely observe and master the physiological response and nursing needs of the children, and provide help to them as much as possible to improve the postoperative comfort and reduce the pain. Hence the quality of life of children could be improved. Based on our results of complications of patients following surgery, there were fewer complications in the study group than in the control group, suggesting that the high-quality nursing based on the concept of childish interest could effectively reduce the incidence of complications in the children after surgery. The results were consistent with Fan et al. [26]. Considering the small sample size of this study, further researches with more participants are warranted to confirm the current findings.

Taken together, the high quality nursing guided by the concept of childish interest elicited beneficial outcomes in reducing the healing time, hospital stay, the pain degree, and incidence of complications, as well as improving the psychological state, the quality of life, and the prognosis of the children harboring cleft lip and palate. It is a valid and alternative method for wide clinical application.

## Data Availability

The datasets generated and analyzed during the current study are available from the corresponding author on reasonable request.
